# Comparison of the Feasibility and Safety of a Deep Sedation Protocol for Pulmonary Vein Isolation With Pulsed Field Ablation, Cryoballoon Ablation, and Radiofrequency Ablation

**DOI:** 10.1161/JAHA.123.033210

**Published:** 2024-05-31

**Authors:** Oskar Marian Galuszka, Thomas Kueffer, Antonio Madaffari, Nikola Kozhuharov, Gregor Thalmann, Helge Servatius, Andreas Haeberlin, Fabian Noti, Hildegard Tanner, Laurent Roten, Tobias Reichlin

**Affiliations:** ^1^ Department of Cardiology Inselspital, Bern University Hospital, University of Bern Bern Switzerland; ^2^ ARTORG Center for Biomedical Engineering Research University of Bern Bern Switzerland

**Keywords:** atrial fibrillation, catheter ablation, deep sedation, pulsed field ablation, Atrial Fibrillation, Catheter Ablation and Implantable Cardioverter-Defibrillator, Electrophysiology

Pulsed field ablation (PFA) is a novel, nonthermal ablation modality that is increasingly used for pulmonary vein isolation (PVI). Despite technical advancements, the procedure remains painful and uncomfortable, requiring adequate patient sedation. Previous studies demonstrated the safety of deep sedation protocols in patients undergoing PVI with cryoballoon (CBA) and radiofrequency ablation (RFA). However, data regarding the feasibility and safety of deep sedation protocols in PFA procedures are scarce^1^, ^2^ and a comparison with CBA and RFA procedures is lacking.

Our aim was to compare the feasibility and safety of a deep sedation protocol in patients undergoing PVI using PFA (Farapulse, Boston Scientific), CBA (Arctic Front, Medtronic), or RFA (CARTO 3, Biosense Webster). The authors declare that all data used for the analyses included in this study are available within the article. Between January 2020 and April 2023, consecutive patients undergoing a first PVI with either of the 3 technologies were analyzed. All ablation procedures were performed using the same predefined deep sedation protocol including midazolam, fentanyl, and propofol administered by a nurse under the supervision of the electrophysiologist.^3^ Procedural patient surveillance included continuous heart rate, peripheral capillary oxygen saturation, and transcutaneous carbon dioxide level monitoring and noninvasive blood pressure measurement at regular intervals, as clinically indicated. Exclusion criteria for the use of this protocol were a body mass index >35 kg/m^2^, severe untreated sleep apnea syndrome, or any other indications requiring intubation and general anesthesia. The study was approved by the local ethics committee and all patients provided written informed consent.

A total of 1049 patients underwent a first PVI using the deep sedation protocol (median age, 67 years [interquartile range, 59–74], 29% female, 59% paroxysmal atrial fibrillation). The distribution among the 3 ablation modalities was PFA in 429 patients (41%), CBA in 412 (39%), and RFA in 208 (20%). Acute procedural success, defined as bidirectional block of all pulmonary veins, could be achieved in 99.7% of the patients (no differences between groups). Procedure duration was higher in the RFA group than in the CBA and PFA groups (RFA 161±55 minutes; CBA 81±29 minutes; PFA 93±41 minutes; Figure [A]). As a consequence, mean total doses of propofol and fentanyl were higher in the RFA group (814±351 mg and 185±61 μg) than in the CBA group (517±213 mg and 139±48 μg) and PFA group (550±204 mg and 157±44 μg; Figure [B] and [C]). After normalization for procedural time, mean doses of propofol and fentanyl per minute were higher in the PFA (6.4 mg and 2.0 μg per minute) and CBA (6.6 mg and 1.8 μg per minute) groups compared with the RFA group (5.2 mg and 1.2 μg per minute; Figure [D] and [E]). Periprocedural conversion to general anesthesia was necessary in 1/427 (0.23%), 2/410 (0.49%), and 0/208 (0%) patients in the PFA, CBA, and RFA groups, respectively (Fisher's exact test: PFA versus CBA *P*=0.485; PFA versus RFA *P*=0.672; CBA versus RFA *P*=0.440). The reasons for conversion were insufficient analgosedation resulting in pain and patient movement in 2 cases and airflow obstruction in 1 patient with chronic obstructive pulmonary disease requiring intubation and mechanical ventilation.

**Figure 1:**
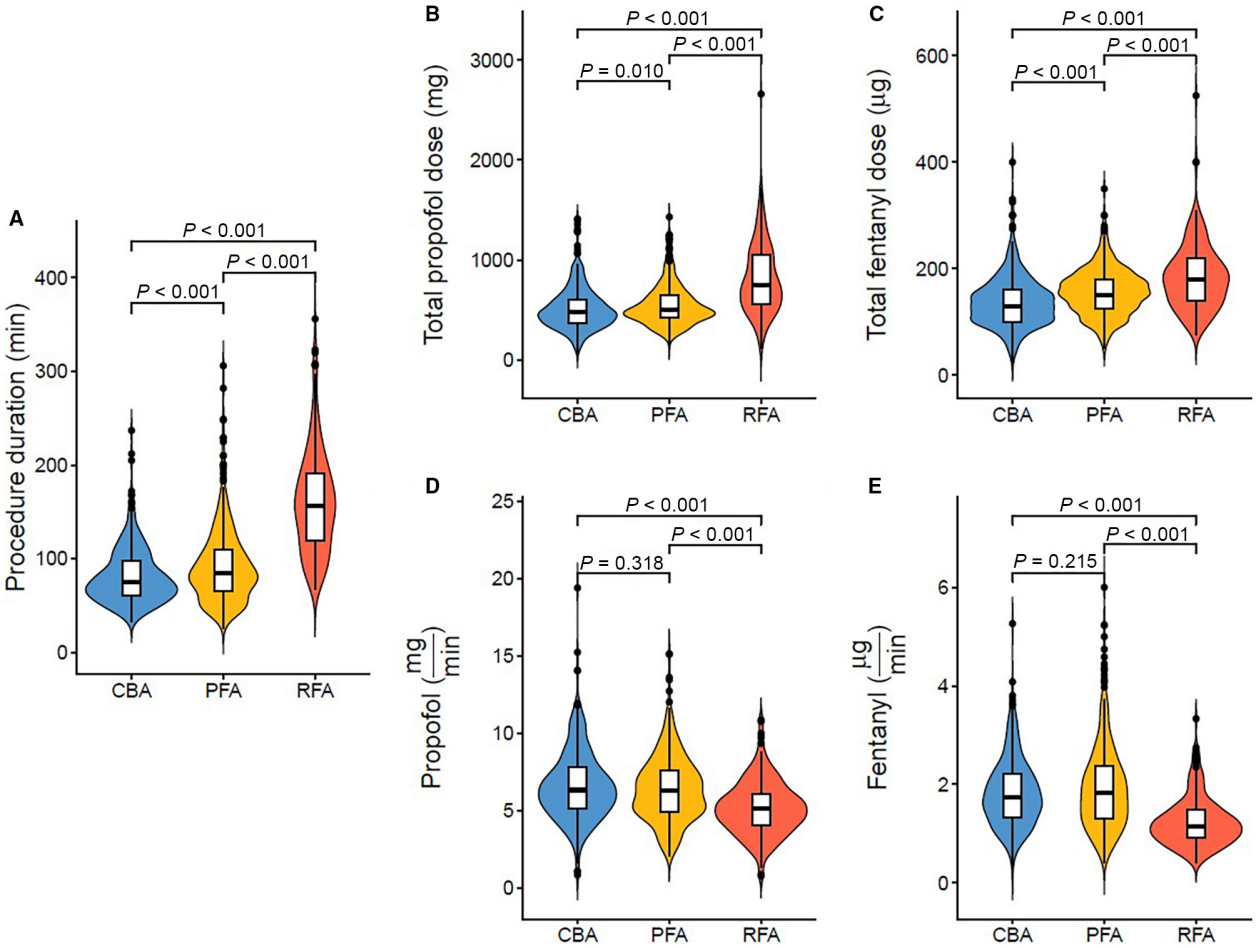
Deep sedation for different ablation modalities. Procedure duration (**A**) and sedatives used (**B** through **E**) during deep sedation. The difference between cryoballoon ablation and pulsed‐field ablation for total sedative dose (**B** and **C**) was not observed when correcting for procedure duration (**D** and **E**). Wilcoxon test was used for group comparisons. CBA indicates cryoballoon ablation; PFA, pulsed‐field ablation; and RFA, radiofrequency ablation.

Our data from 1049 patients indicate that a propofol‐based deep sedation protocol is a safe and suitable sedation strategy for PVI using this particular pentaspline PFA system. After normalization for procedural time, the total doses of propofol and fentanyl required for the PFA procedures were comparable to CBA procedures and higher than in RFA procedures. We believe that this might be indicative of the fact that PFA and CBA are slightly more painful than RFA cases. Sedation complications were very low and comparable to PVI using CBA or RFA. Muscular fasciculations, which are not observed with either CBA or RFA, can occur with PFA and can result in significant patient movement. Although this is not an issue if PFA is used with fluoroscopy only, it will likely result in map shifts in some patients after full integration into 3‐dimensional mapping systems. It will therefore have to be shown if deep propofol sedation can provide sufficient stability for the routine use of 3‐dimensional integrated PFA systems.

Limitations of our data include the following: first, patient satisfaction was not prospectively quantified in our study. However, we are not aware of any negative patient feedback in any of the 3 groups. Second, we performed PFA procedures using only 1 particular PFA device (Farapulse, Boston Scientific). Our findings therefore cannot be applied to other PFA devices. In particular, unipolar rather than bipolar PFA systems and also PFA systems with integrated 3‐dimensional mapping might require different anesthesia protocols.

In conclusion, PVI procedures using this specific pentaspline PFA device can be safely performed using a propofol‐based deep sedation protocol in selected patients under the supervision of the electrophysiologist, thus curtailing the need for general anesthesia for PVI procedures.

## Sources of Funding

None.

## Disclosures

O. Galuszka reports an educational grant from Medtronic. N. Kozhuharov reports research grants from the Swiss National Science Foundation (grant Nr P400PM‐194 477 and grant Nr P5R5PM_210856), Gottfried und Julia Bangerter‐Rhyner‐Stiftung, Freiwillige Akademische Gesellschaft, L. & Th. La Roche Stiftung, and the European Society of Cardiology. A. Haeberlin reports research grants from the Swiss National Science Foundation, Innosuisse, the Swiss Heart Foundation, the University of Bern, the University Hospital Bern, the Velux Foundation, the Hasler Foundation, the Swiss Heart Rhythm Foundation, and the Novartis Research Foundation. He is cofounder and chief executive officer of Act‐Inno, a cardiovascular device testing company. He has received travel fees/educational grants from Medtronic, Philips/Spectranetics, and Cairdac without impact on his personal remuneration. F. Noti reports travel fees, speaker fees, educational grant from Medtronic, Abbott; travel fees, educational grant from Boston Scientific, Philips Spectranetics; and institutional grant from Biotronik, all for work outside the submitted study. L. Roten reports speaker honoraria from Abbott/SJM, consulting honoraria from Medtronic, and research grant to the institution from Medtronic for an investigator‐initiated trial. T. Reichlin reports research grants from the Goldschmidt‐Jacobson Foundation, the Swiss National Science Foundation, the Swiss Heart Foundation, and the sitem insel support fund, all for work outside the submitted study; speaker/consulting honoraria or travel support from Abbott/SJM, Biosense‐Webster, Biotronik, Boston Scientific, and Medtronic; and support for his institution's fellowship program from Abbott/SJM, Biosense‐Webster, Biotronik, Boston‐Scientific, and Medtronic. The remaining authors have no disclosures to report.
